# The COVID-19 pandemic in Vietnam – success, crisis, and endemic: Key thresholds and lessons

**DOI:** 10.7189/jogh.12.03065

**Published:** 2022-11-21

**Authors:** Viet Hoang

**Affiliations:** University of Economics Ho Chi Minh City (UEH), Vietnam

Vietnam successfully controlled the COVID-19 pandemic in the initial periods through public health and social measures. However, the country endured a severe crisis in the next stage and had to change its strategies by focusing on vaccination and stricter measures. As a result, the vaccination rate rapidly increased, and the COVID-19 pandemic has become endemic since April 2022. Two thresholds emerged to change the pandemic into a new stage when indicators and conditions reach certain levels. The main lessons can be withdrawn from Vietnam as follows: collecting sufficient data and information; combining different strategies and suitable measures; identifying thresholds of the pandemics; balancing trade-offs; promoting effective communication; and developing science, vaccine, and vaccination.

## PROGRESS OF THE COVID-19 PANDEMIC IN VIETNAM

COVID-19 is a novel disease and has become one of the most severe health crises in human history with little understanding and high levels of anxiety and panic. The COVID-19 pandemic has caused various health, social, and economic consequences [1]. Controlling the pandemic and reducing its effect requires local, national, and global capacities to prepare and take effective strategies and measures. The success of these measures depends on the organization and financial capacity of public health systems, the coronavirus situation, social and environmental conditions, and political and government systems. These experiences can provide significant lessons for future events.

Though Vietnam was among the first countries affected by the coronavirus from China, it was considered successful in controlling the pandemic in the initial periods and gained the world’s acclaim for its effective strategies and measures, mainly public health and social measures (PHSM) [2,3]. Vietnam reported the first COVID-19 case on January 23, 2020. Since then, it has experienced four waves (Stage 1), a “storm” of the outbreak (Stage 2), and the current stage of an endemic (Stage 3) [3-5]. It could still be successful in controlling the outbreak before wave 4; however, the country endured the COVID-19 crisis at the peak of the catastrophe from June 2021 to March 2022 [4]. During the crisis time, the country ranked the lowest in the Nikkei COVID-19 Recovery Index measured by infection management, vaccine rollouts, and social mobility [6]. Thus, Vietnam changed its strategies and measures to control the crisis by mobilizing all sources to vaccinate the entire population. As a result, the pandemic has been controlled and considered an endemic, and the country has lifted social distancing curbs since April 2022 [5,7].

### Factors of success in the initial periods

Several vital factors determined Vietnam’s success in handling COVID-19 during the early stages, including strict regulations and effective measures, mask-wearing and traveling habits, cautiousness and union, the low number of COVID-19 cases and deaths, and environmental conditions. The government’s successful strategies and measures against COVID-19 could be summarized into detecting, testing, and scaling it up; contact tracing; quarantine; infection prevention and control in health care systems; targeted lockdowns; restrictions of mass gathering, travel, and mobility; and clear and consistent public health messaging and informing [2,3]. A potential hypothesis was that Vietnam could early gain secret information on the danger of COVID-19 and the problematic situation in Wuhan of China; hence the country had prompt preparation and practical measures that were even stronger and sooner than those recommended by the World Health Organization. Notably, vaccination was not widely mentioned as a pivotal contribution to the success, and it gained less attention from the government and people during this time.

### Causes of crisis at the peak of the pandemic

From June 2021, Vietnam was under an uncontrollable situation at the peak of the COVID-19 pandemic, with rapid increases in cases and deaths despite stricter regulations and measures. The PHSM appeared ineffective against the pandemic at this peak. The leading causes of the COVID-19 crisis in this period could be synthesized as follows: (i) the COVID-19 Delta variant could spread more easily and quickly; (ii) Vietnam lacked the data and information on the new variant; (iii) vaccine supply was not sufficient, and vaccination rate was relatively low; (iv) the country was overconfident of the previous successful measures and hesitant about imposing more stringent restrictions and additional measures; (v) there were the shortage of medical equipment and health care systems with the inadequate master plan and preparation; and (vi) the number of total cases and death were very large and new infection ways had emerged, such as goods deliveries, cash transaction, middle tools in a community, and common wind systems in buildings.

**Figure:**
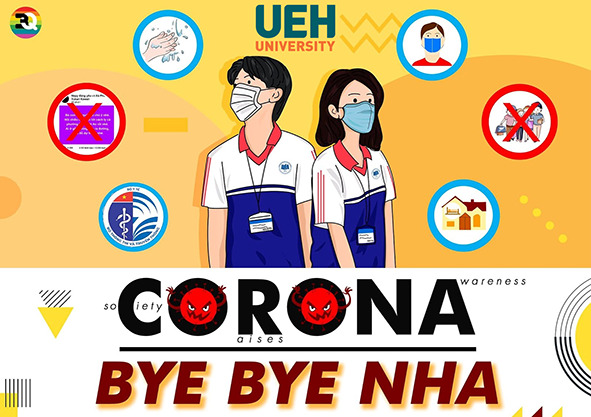
Photo: A communication and propaganda picture of the UEH's guides and determination to fight against the COVID-19 pandemic. Source: The University of Economics, used with permission.

### Vaccination and herd immunity

The COVID-19 crisis caused adverse effects, forced the government to use new measures and focus on vaccines, and changed the public perception of vaccination and health behaviour. As a result, people were willing to vaccinate and accept any vaccines. They also changed to healthier lifestyles, adhered to COVID-19 prevention and restriction measures, and adjusted to the new social and economic situations. On June 25, 2021, Vietnam had a meagre vaccination rate, with total vaccinations per hundred of 3.17 (rank 115th in the world) and fully vaccinated people per hundred of 0.15 (rank 107th in the world). Since then, the vaccination quickly increased, and the country obtained total vaccination per hundred of 134.44 (rank 52nd in the world) and fully vaccinated people per hundred of 57.85 (rank 56th in the world) on December 9, 2021 [8]. Vietnam could receive vaccines from various sources such as COVAX, supports from diplomatic relationships, and purchasing from many countries. In March 2022, Vietnam obtained fully vaccinated people per population of 80% and vaccine boosters per population of 60% [4]. Though cases may increase in some periods, the number of deaths is tiny. Since April 2022, Vietnam’s government has started analysing the situation of COVID-19 to consider it an endemic disease [5].

## THRESHOLDS AND MEASURES

**Figure 1:**
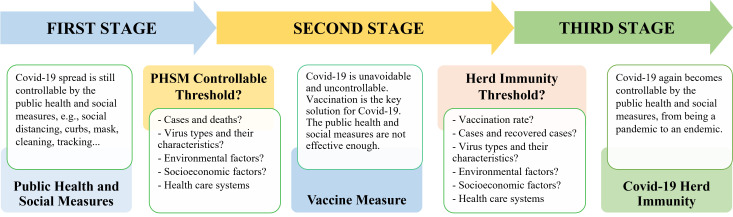
The stages and the thresholds of the COVID-19 pandemic in Vietnam.

The progress of the COVID-19 pandemic in Vietnam can be divided into three stages: the first stage of success in controlling COVID-19 by the PHSM, the second stage of the crisis or uncontrollable situation by the PHSM, and the third stage of becoming endemic. Two thresholds emerged to change the pandemic into a new stage with certain conditions and measures (**Figure 1**).

### Becoming a pandemic: threshold of the public health and social measures

The experiences from Vietnam raise an original and significant question of whether and how the COVID-19 pandemic becomes uncontrollable by the PHSM when total cases, total deaths, and their rates in the population increase up to certain numbers with given factors of social and environmental conditions and coronavirus characteristics. Accordingly, the author proposes the threshold concept of controlling the COVID-19 pandemic by the PHSM and defines it as the marginal levels of the COVID-19 pandemic attributes, where COVID-19 is uncontrollable with given conditions. At the threshold, vaccination is the most effective solution for the pandemic, and the key target is to achieve COVID-19 herd immunity. Therefore, scientists and policymakers need to identify the PHSM threshold and analyse the possibility of herd immunity [9]. What are the attributes of this PHSM threshold? The potential indicators of the PHSM threshold could be environmental conditions, socioeconomic circumstances, total cases, total deaths, cases and deaths in a population, coronavirus types and their characteristics, the capacity of health care systems, and demographic, biological, and lifestyle factors. Until May 2021, Vietnam had only 14 232 cases and 72 deaths. However, from June to September 2021, the pandemic had become highly uncontrollable. On September 7, 2021, Vietnam had total cases of 546 684, daily cases of 22 673, total deaths of 13 701, and daily deaths of 216 with the Delta Variant in central areas, while the full vaccination rate was relatively low, only 3.87% [4,8].

### Moving to an endemic: threshold of herd immunity

Vaccination was the essential and compulsory measure for the COVID-19 crisis when the pandemic reached the PHSM threshold with tremendous rises in cases and deaths. Thus, the vaccination rapidly increased. When the full vaccination rate, total cases, recovered cases, and their rates in population reach certain levels with given factors of social and environmental conditions and coronavirus characteristics, COVID-19 can become an endemic, and herd immunity is achieved. The indicators of the herd immunity threshold can be the full vaccination rate, total cases and cases per hundred, total recovered cases and recovered cases per hundred, coronavirus types and their characteristics, environmental conditions, socioeconomic factors, and health care systems. During the crisis, Vietnam tried to gather as many vaccines as possible and speed up vaccination, and people were required to get vaccinated. As a result, on April 6, 2022, Vietnam had a full vaccination rate of 80.1%, vaccine booster rate of 60.4%, total cases of 10 million, daily cases of 50 337, and daily deaths of 31 with coronavirus type of the Omicron Variant [4,8].

## KEY LESSONS FOR FUTURE EVENTS

The analysis of the COVID-19 progress in Vietnam can result in meaningful experiences for future events, which can be synthesized into vital lessons as follows.1. Collecting sufficient data and information: Vietnam’s initial success in controlling COVID-19 could be based on the prompt and precise information from China. However, in the next period, COVID-19 became a crisis due to the dearth of information about new COVID-19 variants and thresholds. 2.Combining different strategies and suitable measures: COVID-19 is a novel virus and quickly changes with distinct characteristics that require a combination of different strategies and suitable measures.3. Identifying thresholds of the pandemic: The pandemic develops through different stages, and thresholds of changes emerge to separate these stages. Therefore, it is essential to define and estimate the indicators of these thresholds with adequate measures.4. Balancing trade-offs: Policymakers face various trade-offs in response to the COVID-19 pandemic. Their decisions are affected by the pandemic situation, ethical standards, social norms, economic and health benefits, and strategic goals. Therefore, they should choose the right goals and standards, appropriate strategies, and effective measures to maximize benefits and minimize disease burden and losses.5. Promoting effective communication: COVID-19 is a novel disease and one of the most severe health crises in human history with little understanding and high levels of anxiety and fear. Therefore, good communication is critical to managing and overcoming the pandemic. 6. Developing science, vaccine, and vaccination: In the darkest period of the pandemic, the progress of vaccine development is breath-taking and at least five times faster than ever before [10]. When COVID-19 reached the PHSM threshold, vaccines became the most effective and obligatory measure to move it from being a pandemic to an endemic.
